# Engaging community health workers in maternal and infant death identification in Khayelitsha, South Africa: a pilot study

**DOI:** 10.1186/s12884-020-03419-4

**Published:** 2020-11-26

**Authors:** Jude Igumbor, Olatunji Adetokunboh, Jocelyn Muller, Edna N. Bosire, Ademola Ajuwon, Rene Phetlhu, Marjorie Mbule, Agnes Ronan, Fiona Burtt, Esca Scheepers, Kathrin Schmitz

**Affiliations:** 1 School of Public Health, Faculty of Health Sciences, University of the Witwatersrand, Johannesburg, South Africa; 2 mothers2mothers, Cape Town, South Africa; 3 DSI-NRF Centre of Excellence in Epidemiological Modelling and Analysis, Stellenbosch University, Stellenbosch, South Africa; 4 South African Medical Research Council Developmental Pathways for Health Research Unit (DPHRU), School of Clinical Medicine, Faculty of Health Sciences, University of the Witwatersrand, Johannesburg, South Africa; 5 Department of Health Promotion and Education, Faculty of Public Health, University of Ibadan, Ibadan, Nigeria; 6 School of Nursing, University of the Western Cape, Belville, Cape Town, South Africa

**Keywords:** Verbal autopsy, Social autopsy, Death surveillance and response, Death review, Infant mortality, Stillbirth, Neonatal mortality

## Abstract

**Background:**

Engaging community health workers in a formalised death review process through verbal and social autopsy has been utilised in different settings to estimate the burden and causes of mortality, where civil registration and vital statistics systems are weak. This method has not been widely adopted. We piloted the use of trained community health workers (CHW) to investigate the extent of unreported maternal and infant deaths in Khayelitsha and explored requirements of such a programme and the role of CHWs in bridging gaps.

**Methods:**

This was a mixed methods study, incorporating both qualitative and quantitative methods. Case identification and data collection were done by ten trained CHWs. Quantitative data were collected using a structured questionnaire. Qualitative data were collected using semi-structured interview guides for key informant interviews, focus group discussions and informal conversations. Qualitative data were analysed thematically using a content analysis approach.

**Results:**

Although more than half of the infant deaths occurred in hospitals (*n* = 11/17), about a quarter that occurred at home (*n* = 4/17) were unreported. Main causes of deaths as perceived by family members of the deceased were related to uncertainty about the quality of care in the facilities, socio-cultural and economic contexts where people lived and individual factors. Most unreported deaths were further attributed to weak facility-community links and socio-cultural practices. Fragmented death reporting systems were perceived to influence the quality of the data and this impacted on the number of unreported deaths. Only two maternal deaths were identified in this pilot study.

**Conclusions:**

CHWs can conduct verbal and social autopsy for maternal and infant deaths to complement formal vital registration systems. Capacity development, stakeholder’s engagement, supervision, and support are essential for a community-linked death review system. Policymakers and implementers should establish a functional relationship between community-linked reporting systems and the existing system as a starting point. There is a need for more studies to confirm or build on our pilot findings.

**Supplementary Information:**

The online version contains supplementary material available at 10.1186/s12884-020-03419-4.

## Background

Maternal and neonatal mortality is unacceptably high and a global public health concern. Worldwide, about 2.5 million (47%) deaths among children in 2017 occurred in the first month of life [1]. Concurrently, about 295,000 women died during and following pregnancy and childbirth in 2017. The vast majority of these deaths (94%) occurred in low- and middle-income countries (LMIC), with the rural poor most at risk [2, 3]. One longitudinal cohort study (2000–2014) in South Africa showed that the likelihood of maternal death was four times higher in households with low socio-economic status residing in rural areas [4]. Another South African study showed that poorer provinces like rural Free State, had the highest maternal mortality ratio (MMR) (286/100.000 live births) compared to one of the most urbanised provinces, the Western Cape (87/100000) [5]. Despite South Africa’s relative wealth in the region and progressive health policies including free public healthcare for children under 6 years, an expanded vaccination schedule and prevention of mother-to-child transmission (PMTCT) programmes, mortality rates in children younger than 5 years remained high at 37/1000 live births in 2017 [6]. Combating maternal and child mortality requires adequate understanding of the causes of deaths and factors influencing such deaths in order to institute appropriate responses at local levels [7].

Compared to high income countries, many LMICs have unreliable systems to measure and estimate maternal and infant mortality as well as comprehensively capture the causes of death. This is partly due to low investment over the decades in civil registration and vital statistics (CRVS) systems which incorporate formal and non-formal reporting modalities [8]. More than 5 years ago, significant numbers of births and deaths that occurred outside health facilities in most African countries went unreported [9, 10]. These trends have hardly changed with reports showing similar findings from currently existing systems for tracking births and deaths [10]. This is especially the case in children who die at home, constituting over half of deaths in under-fives [11]. Furthermore, in South Africa, limited information remains on healthcare usage patterns, barriers to health care access, delays in care-seeking and quality of healthcare, particularly in poor urban and rural areas [10]. This situation highlights an important gap that requires a more comprehensive and inclusive tracking system for maternal and child health care, that would integrate both facility and non-facility based sources of data [8].

Given this background, there is a need for a functional CRVS system that would augment its data with community based approaches that systematically captures deaths outside the health system. Community based approaches including household surveys, verbal and social autopsy have been recommended in different contexts for identifying maternal and child deaths in the community to support the existing mortality reporting systems [10, 12–15]. Verbal autopsy involves interviewing family members or caregivers of the deceased to identify the likely causes of death and clinical features observed before death [13]. Social autopsy is the process of identifying social, behavioural and health system’s determinants of maternal and neonatal deaths [16].

Involving community health workers (CHWs) in maternal and neonatal care and death review processes in Asia and sub-Saharan Africa (sSA) has reduced maternal and neonatal mortality. CHWs in Bangladesh, Pakistan, India, Kenya, Malawi and Nigeria were demonstrably well positioned, geographically and socially, to deliver essential vital services achieving significant improvements in mother and child health outcomes [17, 18]. The definition of CHWs varies and can include trained lay workers, health volunteers, community health agents, traditional births attendants and community midwives [17]. Studies in sSA including Ethiopia, Malawi and Mali have shown that CHWs in some resource-limited settings have successfully increased the accuracy of population-based maternal and neonatal death estimates through initiatives that link facilities and communities, boosting vital registries and facility records data [15, 16]. Using verbal autopsies, CHWs in Uganda reported that more than half of identified deaths in one community were not recorded in facility-based surveillance, with most of these being among under-fives [18]. To the authors’ best knowledge, no research has reported the use of verbal and social autopsy as part of the existing mortality reporting system in Khayelitsha, which is a partially informal settlement and regarded as one of the largest and fastest growing townships in South Africa. In response to this gap, we conducted a pilot study, using trained CHWs to investigate the extent of unreported maternal and infant mortality in Khayelitsha and explored requirements of such a programme and the role of CHWs in bridging gaps.

## Methods

### Study setting

This study was conducted in Khayelitsha sub-district, located in Cape Town, Western Cape Province of South Africa, with an estimated population of 781,514. Khayelitsha is one of the deprived areas of Cape Town with limited infrastructure and very poor socio-economic indices [19]. There are 16 health facilities – three primary healthcare clinics, five community day centres, two community health clinics, five specialised service centres and one district hospital in the area. In 2017/2018, the maternal mortality in-facility ratio (MMR) was 14.1 per 100,000 live births and the neonatal mortality rate (NMR) was 3.2 per 1000 live births (Western Cape Government, 2018). These figures are lower than the national MMR and NMR according to the South African Demographic Health Survey (SADHS) which are as high as MMR of 536 per 100,000 and NMR 21.1 per 1000 [20].

### Study design

This study employed a mixed methods approach of combining both qualitative and quantitative methods. The quantitative part consisted of case identification of stillbirths, maternal and infant deaths in the sub-district using structured verbal and social autopsy questionnaires. Interviews were conducted with close relatives or associates of the deceased. Qualitative data were collected using different methods such as focus group discussions (FGDs), key informant interviews (KII) and informal conversations that described moments and meanings in individual’s lives – giving cognizance to their lived experiences [21]. To guide the researcher’s application of quality assurance measures, the Consolidated Criteria for Reporting Qualitative Research (COREQ) checklist was utilised [22].

### Participant sampling and data collection

Participants were recruited using a combination of convenience and snowball sampling approach. Ten CHWs who were previously engaged by a non-governmental organization working on HIV-prevention of mother-to-child-transmission of HIV as known as Mentor Mothers (MM), were recruited to work as field workers in this study. MM are women living with HIV who were employed to support other HIV-positive women or mothers and their families. MM provided basic health education and psychosocial support to pregnant women and new mothers on prevention of mother-to-child transmission of HIV and healthy living. For the purpose of this study MM were referred to as CHWs. They were trained for case identification and verbal and social autopsy interviews. They were also trained on research ethical issues relating to confidentiality and sensitivity around deaths, basic counselling, qualitative interviewing skills, data collection, methods to monitor and evaluate field experiences via post-interview reflections, the survey questionnaires and familiarization with the electronic device to capture survey responses. Based on personal experiences of the loss of a neonate, three of these CHWs were selected for a further in-depth interview. The study project manager approached six facility managers from different primary health facilities in Khayelitsha to participate in the study. These managers only acted as mediators and selected three nurses from their facilities for the study. Interviews with those three nurses were conducted in the facility where they were based. When it was agreed to expand the range of interviewees, further interviews with seven key informants were conducted in their respective offices. Key informants were approached to participate through either face-to-face meeting, by telephone or through email. An interview with the study project manager and informal conversations with the CHWs were conducted at the end of the project to understand their experiences during project implementation.

### Community and facility engagement

**Fig. 1:**
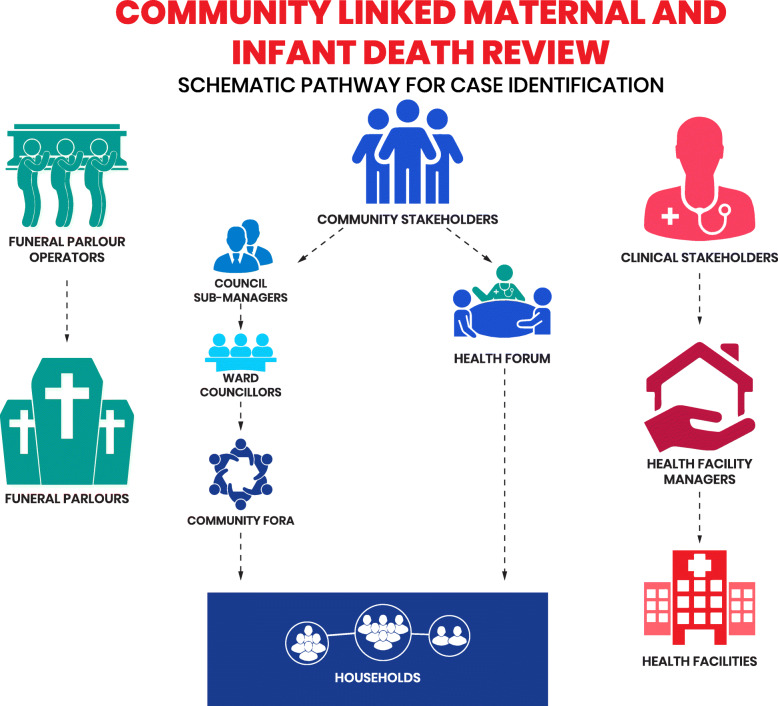
The process of community engagement and case identification

Between May and July 2019, various community stakeholders such as Council Sub-Managers, Ward Councillors, South African National Civic Organization/street committees (groups committed to address the basic needs and aspirations of Khayelitsha residents) and the Khayelitsha Community Health Forum were engaged for their input and advise on fieldwork implementation within the communities. Several clinical managers and funeral service operators were also consulted. Engaging all these stakeholders were required to promote community participation, linkages and collaboration. Figure 1 described the schematic step by step approach adopted with different stakeholders at community level, with the facilities and funeral operators for community engagement and case identification.

### Identification of stillbirths, maternal and infant deaths

This study was designed to identify stillbirths, maternal and infant deaths that occurred within Khayelitsha between January 2017 and July 2019. From June to July 2019, CHWs working in pairs moved around selected areas based on information of deceased cases obtained from street committee members and facilities for case identification. This effort was to identify other cases that the CHWs may not have been aware of. The street committees also nominated community members who were conversant with the area to accompany CHWs while doing door to door household visits (Fig. 1).

For each identified death, CHWs contacted the family and arranged for an introductory meeting. At the initial meeting, they introduced themselves and consoled the family members. The team then gave a description and process of the verbal and social autopsy interviews. The team also identified the most suitable respondent and scheduled an appointment for the interview. Selection criteria for respondents was any relative or family member who was close to the deceased and had knowledge about how they died. Each respondent signed consent prior to the interview. The verbal and social autopsy questionnaire was translated into Xhosa Language, (the language widely spoken in the district). The CHWs used research electronic data capture (REDCap) software installed on a smart phone to conduct interviews and record information concerning the deceased [23]. The team also recorded the interviews on smart phones as backup with permission of the respondents. Data collection tools are attached as Additional files 1 and 2 for maternal and infant death review respectively.

The qualitative interviews spanned a three-months period from May to July 2019. Qualitative and quantitative data collection was conducted simultaneously. One Interview guide was prepared for the FGDs, KII and informal conversation. The guide was pre-tested by CHWs and revisions made i.e. sequencing of questions was adapted to the category of selected key informants. Twelve KIIs and one informal conversation were held. An experienced qualitative researcher conducted the interviews in a private space at a time that was convenient for the interviewees. The ten CHWs who were recruited as fieldworkers for the social and verbal autopsy component of the study participated in a FGD. An informal conversation was conducted with the field workers to capture their field experiences. The process involved three rounds of discussion with 1) specific questions posed at each Table 2) followed by a plenary session to interrogate the responses and 3) lastly with a final round where each person reflected on their personal experience.

### Data analysis

Quantitative data were analysed in Microsoft Excel and results were expressed in frequencies because of the small numbers in the pilot study. Qualitative data analysis was carried out concurrently, where the results of a preliminary analysis from one interview guided the subsequent interview. This stage involved deep listening of the audio files to obtain a general sense of the narratives. The audio files (FGDs and KIIs) were transcribed verbatim by the interviewer. Extracting significant categories from the transcripts relating to the basis of the study were done. The goal was to create categories deductively and thus provide a focus for the next interview. Thematic analysis shows how social, economic and political mechanisms give rise to a set of socioeconomic positions, whereby communities are stratified according to income, education, occupation, gender, race/ethnicity and other factors. These socioeconomic positions in turn shape specific determinants of health (intermediary determinants), reflective of women’s place within social hierarchies.

## Results

### Characteristics of stillbirths, neonatal and childhood deaths

CHWs identified twenty two (22) deaths as follows: 19 neonates and infants and 3 maternal deaths between January 2017 and July 2019. Only 19 deaths, however, could be investigated using verbal and social autopsy guides as follows: 5 stillbirths, 8 neonatal, 4 infant and 2 maternal deaths. No signed consent prohibited investigation of three deaths. More than half of the stillbirths and neonatal deaths (*n* = 9) had death certificates issued. Eight deaths occurred during the neonatal period (Table 1). Majority of deaths occurred in 2019 (*n* = 11), with most deaths occurring in hospitals (n = 11), followed by those that occurred at home (*n* = 4). Almost all mothers of the deceased were still alive (*n* = 16) and more than half (*n* = 9) were living with HIV (Table 1). All respondents who had stillbirths or experienced neonatal deaths were Black African adults.

**Table 1:** Sociodemographic characteristics of the deceased infants and stillbirths with verbal autopsies in Khayelitsha, 2017–2019

CHARACTERISTICS	NUMBERS
**Age at death**	
*Stillbirth*	5
*0–7 days*	5
*8–28 days*	3
*≥ 29 days*	4
**Year of death**	
*2017*	3
*2018*	3
*2019*	11
**Occurrence of place of death**	
*Hospital*	11
*Home*	4
*En-route health facility*	1
*Unknown*	1
**Death certificate**	
*Yes*	9
*No*	8
**Multiple births (twins)**	
*Yes*	1
*No*	16
**Mother still alive**	
*Yes*	16
*No*	1
**Mother’s HIV status**	
*Positive*	9
*Negative*	8
**Underweight at birth (< 2.5 kg)**	
*Yes*	6
*No*	7
*Doesn’t know*	4

The two cases of maternal deaths occurred during 2018 and were Black African women. One was aged between 25 and 34 years with primary school education, while the other, aged 35–44 years, had secondary school education. The families of those two cases were issued death certificates.

### Pregnancy, labour and events after births

Majority of the babies were born in health facilities by skilled personnel (*n* = 12) and 13 were born via vaginal birth. During interviews, one participant (KII10) estimated that *“About 90% of women give birth in facilities*”. Two-fifths of the cases (*n* = 5) had labour and birth complications. Nearly a third of the women developed difficulty in breathing, while five women vomited one time or the other. The deceased women had health problems during pregnancy such as severe bleeding and hypertension. Causes of death were obstructed labour and severe bleeding. Their deaths occurred in health facilities.

### Healthcare utilisation

Healthcare utilisation for cases of stillbirths, neonatal and childhood deaths are summarised in Table 2. Almost all deceased were taken to health facilities for treatment of the illness that eventually led to death (*n* = 15). Only one of those who obtained medical care reported admission problems, two reported poor treatment and three challenges in getting medication.

**Table 2:** Health care utilisation

EVENTS	NUMBERS		
	***Yes***	***No***	***Doesn’t know***
Treatment of illness ^b^	8	6	–
Oral rehydration therapy^a^	1	12	3
Intravenous fluid ^a^	8	7	1
Blood transfusion^a^	0	16	–
Nasogastric tube ^a^	4	11	1
Antiretroviral prophylaxis^a^	4	11	1
Surgery ^a^	0	16	–
Childhood immunisation ^a^	7	9	–
Disclosure of causes of death	8	9	
Health facility visit	15	2	–
Motorised transport to the facility	10	7	–
Problems during admission to the hospital or health facility	1	16	–
Problems with the way (s)he was treated (medical treatment, procedures, interpersonal attitudes, respect, dignity) ^a^	2	14	–
Problems getting medications or diagnostic tests^a^	3	13	–
Any doubts whether medical care was needed in the final days before death	5	12	–
Traditional medicine used in the final days before death	0	17	–

^a^16 respondents ^b^14 respondents

### Perceived causes of maternal and child illnesses and deaths in Khayelitsha

During verbal and social autopsies, CHWs explored the causes of deaths of both maternal and infant deaths. Most deaths in facilities were attributed to health system issues such as poor quality of care and long queues and waiting time:

*“After birth they didn't come, the nurses didn't help me, and the colouring of my body changed green after that I collapsed.”* (*N011, infant death, respondent: mother of the deceased).*

*“The patient passed away before she was helped.”* (*M001, maternal death, respondent: relative of the deceased).*

In other instances, these factors demotivated women from attending clinics, thereby risking their deaths:

*“The quality of the health system is poor. The health system is failing women. There are long queues. The waiting time is a factor that demotivates women from attending the clinics.” (KII10, respondent works in the field of maternal deaths reporting)*

Furthermore, staff shortages compounded by poor attitudes from providers was also a factor contributing to neonatal and infant deaths:

*“They did not give the baby attention, they just told me to put the baby on top of the bed and put something on top.”* (*N005, infant death, respondent: mother of the deceased).*

Secondly, most interviewees reported various contextual and socio-cultural factors linked to maternal, neonatal and infant deaths. Poverty and unemployment were the primary challenge in the community. This was closely discussed alongside poor housing, poor access to basic sanitation and water supply, solid waste accumulation and crime. The excerpt below exemplifies lack of access to basic sanitation:

*“Where I live, there are no toilets. We have communal plastic toilets that are used by everybody and it isn’t always clean.”* (*CHW, KII participant*).

A health worker stated that neonates and infants from informal settlements were at higher risk during ‘*diarrhoea season’*, when shortage of water and sanitation have a multiplier effect on health risks within informal settlements. Another health worker concurred when she said:

*“The area is not safe for the kids; the hygiene is bad. There are many cases of diarrhoea due to lack of basic services” (KII09, Member of Khayelitsha community health forum)*

Crime also attributed to poor health and deaths especially when it hindered health workers from doing their work: *“Khayelitsha is the crime capital of the world!”* (Sub-district ward council member, KII07 participant) while another participant concurred: “*There are a lot of breaking-in, we lose computers which affects how we work. It makes seeing patients hard and takes longer.” (KII06, clinical nurse).*

Participants also cited how stigma, especially for HIV, influenced women’s access to care and disclosure of their status:

*“[The] mother feared to go to the hospital because one of her neighbours works at the clinic. She stayed at home, her child died” (KII06, clinical nurse)*

Further, cultural beliefs were also reported as contributors to deaths:

*“People suspected that it was a bad spirit, as they didn't know at the hospital what was coming out of his mouth, so the elders suspected witchcraft.”*(*N001 infant death, respondent: relative of the deceased*).

Third, interviewees identified factors that were linked with individual characteristics such as lack of education and people’s risky behaviours which were said to influence maternal and infant deaths:

*“Some pregnant women are careless; they don’t go to the clinic. This is not about access but people just being careless.” (KII08, funeral parlour attendant)*

Some factors such as women’s health status also determined the health status of the unborn baby. For example, women with hypertension were said to be at risk of losing their children.

*“Hypertensive state, when its high, can cause deaths. I remember there was a lady who was having convulsions. She was pregnant and she passed away in the ward. When checked [by clinician], they said her hypertension was high.” (KII05, Mentor Mother)*

*“My child died because I was stressed a lot. Even if the baby was going to be alive, he would not be ok because I was stressed a lot.” (KII04, Mentor Mother)*

### Death tracking and review process

During interviews with the experts on death reviews, it was revealed that the confidential enquiry into maternal deaths has been operational in South Africa for over a decade. Health workers were conversant with the procedure of notifying deaths that occurred in the hospital.

*“The health worker notifies death […] fills a form, which gets collated with other death reports…so countrywide information is available on number of deaths and causes”. (KII10, respondent works in the field of maternal death reporting)*

However, this death review tracking system was only applicable for the patients who died in facilities rather than those who died in the community. Although participants suggested the existing reporting system to be linked to the community, it appeared that deaths that happened in the community were not linked to health facilities.

*“The deaths that are recorded are the ones if the child was brought here and died in the facility. That is the death that will be reported, and the statement will be written on what happened.” (KII06, clinical nurse)*

*“Deaths that happen in the community don’t get reported.” (KII09, Member of Khayelitsha community health forum)*

### Reasons for unreported maternal and infant deaths

Most interviewees reported various reasons why deaths went unreported in the civil registration system. Reasons such as lack of links between facility and community, a fragmented healthcare system and social-cultural issues including a culture of blame with stillbirths and late abortions, were widely discussed.

First, no proper established links existed between facilities and community. This made it difficult to capture all deaths happening outside facilities:

*“There is a weak link in the system […] community aspect isn't so well developed. We don’t have a proper way of reporting from the community. For example, patients might be brought to hospital by an ambulance, but nobody knows what happened before [at home]. What is lacking is interviewing the relatives before a patient left home…” (KII10- works in the field of maternal deaths reporting)*

As a result, many interviewees believed mortality rates, especially for infants to be higher than reported.

*“We have no way of knowing about the deaths that occur outside facilities, but we do know from the mortuaries that there are more babies that die in the first month than we have recorded.” (Senior health official, KII participant).*

Secondly, a fragmented death reporting system also contributed to higher numbers of unreported deaths.

*“Mortuary attendants sometimes don’t know what happened after post-mortem, the information comes sealed as confidential and goes to the hospital.” (KII08 funeral parlour attendant)*

*“Most mortuaries have higher numbers than what is at the facility. Some infant deaths are taken to mortuary without passing through the health facility.” (KII10 works in the field of maternal deaths reporting)*

In addition, disjointed services for mother and child clinic made death review difficult.

*“Difficulties arise when mother and baby go to different clinics. That makes tracing difficult. I don’t know what can be done.” (KII06 clinical nurse)*

Moreover, lack of proper documentation in facilities worsened the link between facility and community data.

*“District health committees are not so well organised. District health facilities must know more about death reviews and how the system works but this is not happening…” (KII10, respondent works in the field of maternal deaths reporting)*

*“Also from our side (community), we have never asked this information from the facility. I think we only focus on [primary health] facility issues and leave the maternal obstetric units [MOUs – delivery units run by midwives] aside. There are a lot of complaints but we have not discussed issues around maternal or infant deaths. This is because, a space was never opened for us to interrogate this.” (KII09, Member of Khayelitsha community health forum)*

Third, issues around socio-cultural factors and practices associated with deaths were also major contributors of unreported deaths. For example, interviewees revealed that deaths involving stillbirths and infants were mostly buried the same day in the community to avoid mortuary costs. Stillbirths were not reported to the facility but were buried in the community:

*“Most stillbirths occur at home and we don’t register stillbirths.”(KII10, respondent works in the field of maternal deaths reporting)*

In addition, maternal deaths associated with unsafe abortions were not reported. Participants narrated that they would hardly disclose the cause of such deaths for fear of police arrests:

*“Most families don’t report deaths that occur as a result of abortions. Even if the police comes, the family will not reveal the “real” cause of death of the women” (KII05, Mentor Mother)*

Furthermore, migration patterns within the informal settlement made it difficult to track or record deaths:

*“Some mothers are moving from place to place. They come in the facility without proper records. If we don’t have their records, it’s difficult to track what happened before they came*.” (KII06, clinical nurse)

### CHW’s experiences and perspectives on conducting verbal and social autopsies

An informal conversation with the CHW team and KII with the study project manager captured fieldwork perspectives and experiences. This identified design and implementation gaps in this study and lead to lessons to support the operationalisation of future projects.

### Gaining access to the community

The most effective way to foster community involvement was to allow the existing community structures to develop their own agenda concerning implementation of the community linked death review reporting system. While the implementing agency regarded the CHWs as ‘insiders’ because they were residents of Khayelitsha, they were simultaneously perceived as ‘outsiders’ beyond the confines of the facilities. A few difficulties reported by the project manager when entering the field as ‘outsiders’ included:

*“The CHWs were not ‘known’ by the local leadership and impeded access to potential interviewees.”*

*“The local leadership insisted that opportunities be created for local community members to be included in the study team as community informants.” (KII11, project manager)*

CHWs agreed that all training sessions they participated in, were useful and relevant to their work. One participant said: *“the training has given me new skills on interviewing, and tools to do my job well’.* This increased their confidence to utilise these skills and conduct interviews. However, CHWs had challenges in completing the post-interview reflections (PIRs) and conducting debriefing sessions, both of which were integrated into the project design as quality assurance tools for monitoring, evaluating and responding to field experiences. They suggested that future training should be adjusted to include more opportunities for role-playing, debriefing and writing up field notes and PIRs. CHWs also suggested a need for establishing teamwork communication and support strategies such as establishing *WhatsApp* groups, sharing each other’s contact phone numbers, daily and weekly debriefing meetings to talk about field experiences and to plan activities.

Recruiting participants into the study was a challenging task that required higher levels of effort than anticipated. CHWs reported that the number of cases found in Khayelitsha were low overall, but more cases were in fact identified than were recorded in facilities. These cases mainly came from informal settlements, and CHWs were convinced that additional cases could be found if the project had a longer timeframe. While compensation also involves several ethical principles, justice and respect, CHWs felt that appreciating the time and effort of participants was valuable and worthy of recognition by giving a little token, such as food vouchers since most respondents were still grieving and unemployed.

Lastly, CHW observed that interviewees felt more comfortable with discussions when they were no longer recording. Communication tended to be less formal, more sociable and more spontaneous – this is when they got the *‘real story’*. Even though participants consented to have the interview recorded, interactions that took place when they were being recorded were less relaxed. Strategies such as having multiple engagement with participants, especially before interviews take place, or conducting several interviews with the same participant were suggested. Open ended questions should be answered at the beginning of the interview.

### Strategies and recommendations in improving death review process

Participants cited various strategies that could improve establishing a link between the community and facility, thereby improving death review processes (see summary in Additional file 3). First, it was mentioned that establishing community links and working closely with CHWs would be beneficial in getting an update about what happens in the community:

*“There is need to have a grass-root system that would identify deaths and report them. CHWs are good but overwhelmed” (KII10, respondent works in the field of maternal death reporting)*

*“We as the community we need to do monitoring and link with the hospital to understand why a mother lost her baby at the 11th hour…” (KII09, Member of Khayelitsha community health forum)*

However, linking community and facility in dialogue about deaths at community level appeared a thorny issue that needed serious thought on how to go about it.

*“There is a need to come up with ways of having meetings after death that is community- facility meetings. The challenge is how to harmonise the meetings during the period of anger, when community might be blaming the facility.” (KII10, respondent works in the field of maternal death reporting)*

Educating the community about death review processes was said to be an important step because issues related to deaths are always emotional and it would be better if community members understood the importance of conducting death reviews. This was also said to be helpful in facilitating community entry and sensitizing individuals to go to hospital for treatment.

*“There is need to have a dialogue… …a joint discussion that might lead to a kind of activism. Involving community around deaths is something that needs to be thought through” (KII07, respondent is a counsellor, Khayelitsha training centre)*

It was also recommended that families need to be educated in health matters as well as to work closely with health system authorities or staff especially in notifying death incidences that happen at home:

*“To the families, if someone has passed away, they must report to the facility. It really depends on the families to go to the hospital than just going to the funeral parlour.” (KII08, funeral parlour attendant)*

*“Those people working in the hospital should have some time to go to the community and educate women. Cultural issues such as man is the head of the house and must be listened to is a problem. For example, a man can tell a woman not to go to the clinic because it’s against the culture” (KII04, Mentor Mother)*

## Discussion

Our results demonstrate that CHWs are able to conduct quality verbal and social autopsy in low income settings such as Khayelitsha. Our findings conform with the patterns and causes of maternal and infant mortality which have been reported in South Africa [11, 21]. Importantly, we could show differences in the causes of deaths between those occurring in healthcare facilities and those in the community.

Maternal and infant deaths in facilities were attributed to health system issues such as poor quality of care, staff shortages and provider attitudes. Similar findings have been reported elsewhere [11, 24]. Parents and relatives of the deceased in the communities were dissatisfied with the healthcare system. Many blamed the nonchalant attitudes of healthcare workers in the facilities as cause of death of their relatives. Deaths at community level were attributed to contextual and socio-economic factors. It is therefore important to understand both health system as well as community factors linked to death to come up with contextually appropriate strategies to reduce maternal and infant deaths.

Although there was a functional civil registration for maternal and infant deaths, this only captured deaths in the health care system. Considerable numbers of deaths, however, occurred at community level, yet these numbers were not captured due to poor links between the community and healthcare system. This gap may be exacerbated by a fragmented death reporting system where health facilities did not know what was happening in the mortuaries and vice versa. Data from healthcare facilities need to be merged across different contexts in South Africa to inform national mortalities, leading to underestimation of the number of true deaths in the community. Earlier studies in South Africa reported that most deaths that occur outside health facilities go unreported [10, 25]. Thus, the number of unreported deaths which were identified using verbal and social autopsies indicate a strong need to better understand health seeking behaviour of the general population, especially amongst the vulnerable population of under-fives. Data from verbal and social autopsies could augment health facility data, especially with respect to identification and improving the death reporting system in South Africa [15, 16].

Verbal and social autopsies have been recommended to augment civil registration system in death review processes. The process, however, requires involvement of several stakeholders with feedback at every step [26]. Our study shows that CHWs can successfully conduct verbal and social autopsies while engaging with various stakeholders at community level. This is because CHWs were proficient in using the local language, had good skills in conducting interviews, basic knowledge of medical terminologies and could use electronic devices for data collection. CHWs also had skills in interpersonal communication, empathy and patience which were essential in conducting interviews on sensitive subjects. Smooth running of verbal and social autopsies needs development of proper links between community and facilities through dialogue and this would enhance trustworthiness and transparency of death review processes. It was also recommended that community members need to be educated about their role as key players in death review processes.

### Limitations

This was a pilot study, conducted within a short period frame and thus data may not be comprehensive enough to make appropriate conclusions concerning reporting of deaths or to adequately engage in concrete feedback from community stakeholders on issues pertaining to infant and maternal deaths in Khayelitsha. In addition, the team could not gain access to some communities due to security concerns and ongoing conflicts among street committees. Protracted engagement with community stakeholders delayed field work implementation and reduced the number of weeks available for case identification and interviews. Our findings, however, are in line with other studies that have delved into in-depth social and verbal autopsies [24]. Findings can be used to design a larger study with an aim of addressing these limitations.

### Recommendations

Community engagement and advocacy for improving policies are essential requirements for success of similar community linked death reviews. Key stakeholders are very essential for gaining access to every part of the sub-district and ensuring security of interviewers [26]. Stakeholders like ward councillors, street committee members and health fora were engaged in order to gain access to households. There is also need for intersectoral collaboration and coordination by working with community and facility stakeholders [13, 27]. This is made possible by defining joint activities, health service provision and advocacy, as well as maximising resources and impact through coordinated, collaborative activities in the context of community networks and partnerships.

Training and provision of human resources with appropriate personal, technical and organisational capacities; financing resources including operational and core funding; material resources, including infrastructure, information and essential commodities such as medical and other products and technologies are important [25]. Processes of organisational and leadership strengthening, monitoring and evaluation should also be in place [25]. This study recruited field workers who are members of the community, trained to conduct interviews within the context of the project and monitored throughout the study period. Essential kits like smartphones, bags, and writing materials were provided for field workers. Likewise, the implementing agency also provided financial and operational supports.

Policymakers and implementers in conceptualising community-linked death review programmes should establish functional relationships between the community-linked reporting system and the existing system. Involving community members in tracking and documenting every death in the community is essential in identifying and reporting all deaths within the communities. Engaging the key stakeholders from the highest to the lowest level of the community structure to capture every death is a necessity [24]. There is a need to create time for regular reviewing of cases with the healthcare facilities and public health services and data personnel for validation in order to improve the reporting process. Real time reporting and verbal autopsy for deaths that did not occur in health facilities by appointed CHWs should be implemented in South Africa.

## Conclusion

Verbal and social autopsies are important tools in death reviews that can augment data captured by CRVS. This pilot study showed the possibility to use CHWs to track and conduct verbal and social autopsy at community level. CHWs viewed their roles in a positive light and felt good about helping others in similar situations to their own. Establishing an enabling environment, ensuring intersectoral collaboration and coordination, resources and capacity building, organisational and leadership strengthening, and monitoring, evaluation and planning are essential for a successful community-linked death review system. Overall, we believe that engaging CHWs in death reviews represents a promising and likely cost-effective means of improving measurement of vital statistics in resource constrained settings and mobilising stakeholders to address causes of death in the community.

## Supplementary Information

**Additional file 1.** Maternal death record review form.

**Additional file 2.** Infant death record review form.

**Additional file 3.** Summary of community-linked death review components.

## Data Availability

The data that support the findings of this study are available from the senior author, KS, upon reasonable request. It is not made publicly available due to the sensitive nature of mortality studies and in particular this pilot study with a small sample size. The data collection tools are added to this publications as supplementary documents.

## Abbreviations

ANC: Antenatal care
CDSR: Community-based death surveillance and response
CHWs: Community health workers
CRVS: Civil registration and vital statistics
FGD: Focus group discussion
LMICs: Low-and middle-income countries
NMR: Neonatal mortality rate
KII: Key informant interview
MMR: Maternal mortality rate
MM: Mentor mother
PIR: Post-interview reflections
PMTCT: Prevention of mother-to-child transmission of HIV
RAMOS: Reproductive age mortality study
SA: Social autopsy
VA: Verbal autopsy
VASA: Verbal autopsy and social autopsy
