# Supplementary material for: Engaging community health workers in maternal and infant death identification in Khayelitsha, South Africa: a pilot study
Source: BMC Pregnancy Childbirth. 2020 Nov 26;20:736. doi: 10.1186/s12884-020-03419-4 (PMC7690162; doi:10.1186/s12884-020-03419-4)
Supplement: Supplementary file 1 — Additional file 1. Maternal death record review form. [file 12884_2020_3419_MOESM1_ESM.pdf]

Section 1 Maternal Record

Study ID ______________________________

section_1a_information_on_maternal_death_responden

A1 Initials of verbal autopsy respondent ______________________________

A1a Location of respondent
- ◯ Khayelitsha
- ◯ Nkanini
- ◯ Zwezwe
- ◯ Kuyasa
- ◯ Harare
- ◯ Litha Park
- ◯ Site B
- ◯ Site C
- ◯ Mkhaza
- ◯ Makhaya
- ◯ Mandela Park
- ◯ Town Two
- ◯ Green Point
- ◯ Ekuphumleni
- ◯ Graceland

A2 What is your/the respondent's relationship to the deceased?
- ◯ Spouse
- ◯ Father/Mother
- ◯ Sibling
- ◯ Parent in Law
- ◯ Sibling in Law
- ◯ Neighbour
- ◯ Son/Daughter
- ◯ Son/Daughter in Law
- ◯ Uncle
- ◯ Aunt
- ◯ Cousin
- ◯ Nephew
- ◯ Niece
- ◯ Grandfather/mother
- ◯ Other relative (specify)

A2a Specify relationship if other ______________________________

A3 Gender of the respondent
- ◯ Male
- ◯ Female

A4 Did you/the respondent live with the deceased in the period leading to her death?
- ◯ Yes
- ◯ No

A5 Were you present with the deceased at the time of death
- ◯ Yes
- ◯ No

section_1b_personal_details_of_the_deceased

B0 What was the initials of the deceased?

____
(Initials only)

B1 On what date was (NAME) born?

____
(Indicate 01-01-1900 if respondent does not know)

B2 On what date did (NAME) die?

____
(Indicate 01-01-1900 if respondent does not know)

B3 Where did (NAME) die?

- ◯ Husband home
- ◯ Mother/father's home
- ◯ Neighbour/relative's home
- ◯ At health facility
- ◯ On the way to health facility
- ◯ Others (Specify)

B3a Specify if place of death is other

____

B4 How old was (NAME) when she died (in years)

____
(Indicate 999 if respondent does not know)

B5 What was (NAME's) race

- ◯ Black African
- ◯ White
- ◯ Coloured
- ◯ Indian
- ◯ Asian
- ◯ Other (Specify)

B5a Race if other was specified

____

B6 What was (NAME's) highest level of schooling?

- ◯ Primary
- ◯ Secondary
- ◯ Tertiary
- ◯ None
- ◯ Don't Know

B7 What was (NAME's) occupation, that is, what kind of work did she mainly do?

- ◯ Employed
- ◯ Unemployed
- ◯ Self-employed
- ◯ House wife
- ◯ Student
- ◯ Others (specify)
- ◯ Don't Know

B7a Specify other occupation

____

B8 What was (NAME's) marital status at the time of death?
- ◯ Never married
- ◯ Married
- ◯ Widow
- ◯ Divorced
- ◯ Co-habiting
- ◯ Unknown

section_1c_pregnancy_information

C1 Please tell me about (NAME's) health in the six months before she became pregnant this time

____
(PROMPTS Health problems, illnesses, operations, medications)

C2 How many times had (NAME) been pregnant in total?

____
(Indicate 99 if respondent does not know)

C3 Was there any complications in the previous pregnancies?
- ◯ Yes
- ◯ No
- ◯ Don't know
- ◯ Refuse to answer

C3a Please list/name the complication/s

____

C4 How many of these pregnancies resulted in a live born baby?

____
((If no live birth write "0"), 99 if respondent does not know)

C4a How many live births were males?

____
(Indicate 99 if respondent does not know)

C4b How many live births were female?

____
(Indicate 99 if respondent does not know)

C5 How many of these live born babies are still alive? (If no living child write "0")

____
(Indicate 99 if respondent does not know)

C5a How many live male born are still alive?

____
(Indicate 99 if respondent does not know)

C5b How many of the live female born are still alive?

____
(Indicate 99 if respondent does not know)

C7 Infant status:	◯ Still birth<br>◯ Died immediately after birth<br>◯ Alive 1-7 days<br>◯ Alive 8-28 days<br>◯ Alive after 28 days<br>◯ Pregnancy was terminated<br>◯ Unknown<br>(The condition of the infant that was delivered)
C6 How many stillbirths did she have in her lifetime? (If no stillbirth write "0" in total)	______________________<br>(Indicate 99 if respondent does not know)
C6a How many male stilbirths? (Indicate "0" if none)	______________________<br>(Indicate 99 if respondent does not know)
C6b How many female stillbirths? (indicate "0" if none)	______________________<br>(Indicate 99 if respondent does not know)
C8 Please tell me about (NAME's) most recent pregnancy	___________________________<br>(Health problems (e.g: bleeding, fever, convulsions), illnesses operations, medications?)
C9 Where did (NAME) deliver (last pregnancy)?	◯ Home<br>◯ PHC/ Clinic<br>◯ Tertiary Hospital<br>◯ Private hospital<br>◯ District hospital<br>◯ In-Transit<br>◯ Any other place(specify)
C9a Specify name of delivery facility if other	______________________
C10 When did (NAME) start antenatal care during this pregnancy? (Age of pregnancy in months)	______________________<br>(Indicate 99 if respondent does not know, 98 if did not attend ANC)
C11 How many times did she attend antenatal care during this pregnancy?	______________________<br>(Indicate 99 if respondent does not know)
C12 How many months pregnant was (NAME) when she went into labour?	______________________<br>(Indicate 99 if respondent does not know)
C13 Who conducted the delivery- if at home or in health facility	◯ Trained midwife<br>◯ Doctor<br>◯ Nurse<br>◯ Other (specify)
C13a Specify staff who conducted delivery if other	______________________

C14 Type of delivery	◯ Normal<br>◯ Caesarean<br>◯ Assisted
C15 Outcome of delivery	◯ Live Single<br>◯ Live Multiple<br>◯ Stillbirth<br>◯ Other
C15a Other outcome of delivery	__________________________
C16 Please tell me about (NAME's) delivery (if any health problems)	◯ None<br>◯ Prolonged labour<br>◯ Heavy bleeding<br>◯ Delayed/retained placenta<br>◯ Fever<br>◯ Others (specify)<br>(PROMPTS: Health problems during delivery)
C16a Other delivery detail if any problem	__________________________
C17 Please tell me about (NAME's) health problems following delivery (if applicable)	◯ Bleeding<br>◯ Fever<br>◯ Convulsions<br>◯ Offensive Vaginal discharge<br>◯ Hypertension<br>◯ Others (specify)<br>(PROMPTS: Health problems (e.g: illness, operations, medications) where she went for postnatal care, who attended her during postnatal care?)
C17a Other postnatal health problem	__________________________
C18 Please tell me about the health problems in the last 7 days of (NAME's) life	◯ Bleeding<br>◯ Fever<br>◯ Convulsions<br>◯ Offensive Vaginal discharge<br>◯ Hypertension<br>◯ Others (specify)<br>(PROMPTS: Health problems (e.g: illness, operations, medications) where she went for postnatal care, who attended her during postnatal care?)
C18a Other health problems in the last 7 days of life	__________________________
C19 Name and location of nearest government/private facility providing health services	________________________________
C20 How many minutes does it take to get to the above facility from the residence.	__________________________<br>(Time in minutes. Indicate 99 if unknown)

C21 Number of institutions visited before death

____
(Indicate 99 if respondent does not know)

C21a List in order from the first to the last if >1 institution was visited

____

C22 How many hours did it take between identifying the problem and deciding to seek care

◯ < 1 hour
◯ 1- < 6 hours
◯ 6-24 hours
◯ > 24 hours
◯ Does not know

C23 Please tell me what happened between identifying the problem and deciding to seek care?

____
(PROMPTS: Who made the decision, any delay in making the decision, reasons for delay. Other health care visits (e.g. TBA, sing'anga))

C24 How many minutes did it take to get to the health facility?

____
(Indicate 99 if respondent does not know)

C25 Please tell me about the journey to the health facility?

____
(PROMPTS: How did she travel, any delay in getting to the health facility, reasons for delay)

C26 How many minutes did it take to receive care after arriving at the health facility?

____
(Indicate 99 if respondent does not know)

C27 Please tell me about the care (NAME) received at the health facility?

____
(PROMPTS: Any delay in receiving care, reasons for delay Was there enough staff, drugs, equipment, was she treated with respect?)

C28 Please tell me the factors that you think contributed to (NAME's) death

◯ Severe bleeding
◯ Prolonged and obstructed labour
◯ High blood pressure/convulsion
◯ Termination of pregnancy
◯ Severe infection
◯ Other (specify)
(PROMPTS: Factors which contributed to her poor health, individual factors, family factors, community factors, health facility factors)

C28a Specify other factor contributing to death

____

C29 Please tell me how you think women could be prevented from dying in future

____
(PROMPTS: Strategies for women, families, communities, health facilities)

C30 Is there anything else you would like to add?

____

C31 Did (NAME) attend postnatal care clinic

- ◯ Yes
- ◯ No
- ◯ Doesn't know
- ◯ Refused to answer

C32 What caused (NAME) death

- ◯ Severe bleeding
- ◯ Hypertension/Convulsion
- ◯ Prolonged/obstructed labour
- ◯ Severe bleeding
- ◯ Others (Specify)
- ◯ Doesn't know

C32a Other cause of death

____

C33 - Where did the (NAME) die?

- ◯ Hospital
- ◯ Other health facilities
- ◯ Home
- ◯ Enroute health facility
- ◯ Others (specify)
- ◯ Doesn't know
- ◯ Refuse to answer

C33a Other place of death

____

C35 Did the family obtain a death certificate for the deceased.

- ◯ Yes
- ◯ No
- ◯ Don't know
- ◯ Refuse to answer

section 1d: Indirect Cause of Death -history_of_injuries_and_accidents

D1 Did she suffer from any injury or accident that led to her death?

- ◯ Yes
- ◯ No
- ◯ Doesn't know
- ◯ Refused to answer

D2 Was it a road traffic accident?

- ◯ Yes
- ◯ No
- ◯ Doesn't know
- ◯ Refused to answer

D3 Was she injured in a non-road transport accident?

- ◯ Yes
- ◯ No
- ◯ Doesn't know
- ◯ Refused to answer

D4 Was she injured in a fall?	◯ Yes<br>◯ No<br>◯ Doesn't know<br>◯ Refused to answer
D5 Was there any poisoning?	◯ Yes<br>◯ No<br>◯ Doesn't know<br>◯ Refused to answer
D5a What was the poison?	__________________________________<br>(This refers to substance that caused the poisoning e.g insecticide)
D6 Did she die of drowning?	◯ Yes<br>◯ No<br>◯ Doesn't know<br>◯ Refused to answer
D7 Was she injured by a bite or sting by venomous animal?	◯ Yes<br>◯ No<br>◯ Doesn't know<br>◯ Refused to answer
D7a Name of animal that bite or stung the child	__________________________________
D8 Was she injured by burns/fire?	◯ Yes<br>◯ No<br>◯ Doesn't know<br>◯ Refused to answer
D9 Was she a subject of violence (suicide, homicide, abuse)?	◯ Yes<br>◯ No<br>◯ Doesn't know<br>◯ Refused to answer
D9a Type of violence	__________________________________
D10 Was the mother injured by a force of nature?	◯ Yes<br>◯ No<br>◯ Doesn't know<br>◯ Refused to answer
D10a which force of nature injured the child?	__________________________________
D11 Was it electrocution?	◯ Yes<br>◯ No<br>◯ Doesn't know<br>◯ Refused to answer
D12 Was the injury accidental?	◯ Yes<br>◯ No<br>◯ Doesn't know<br>◯ Refused to answer

D13 Did she encounter any other injury?	◯ Yes<br>◯ No<br>◯ Doesn't know<br>◯ Refused to answer
D13a Name of other injury	__________________________________
D14 Was the injury intentionally inflicted by someone else?	◯ Yes<br>◯ No<br>◯ Doesn't know<br>◯ Refused to answer
D14a who inflicted the intentional injury?	__________________________________
