# Supplementary material for: Engaging community health workers in maternal and infant death identification in Khayelitsha, South Africa: a pilot study
Source: BMC Pregnancy Childbirth. 2020 Nov 26;20:736. doi: 10.1186/s12884-020-03419-4 (PMC7690162; doi:10.1186/s12884-020-03419-4)
Supplement: Supplementary file 2 — Additional file 2. Infant death record review form. [file 12884_2020_3419_MOESM2_ESM.pdf]

Section 2 Neonatal Record

Study ID ______________________

section_2a_information_on_neonatal_death_responden

A1 Initials of verbal autopsy respondent ______________________

A1 Location of respondent
- ◯ Khayelitsha
- ◯ Nkanini
- ◯ Zwezwe
- ◯ Kuyasa
- ◯ Harare
- ◯ Litha Park
- ◯ Site B
- ◯ Site C
- ◯ Mkhaza
- ◯ Makhaya
- ◯ Mandela Park
- ◯ Town Two
- ◯ Green Point
- ◯ Ekuphumleni
- ◯ Graceland

A2 What is your/the respondent's relationship to the deceased?
- ◯ Parent
- ◯ Other family member
- ◯ Friend
- ◯ Health worker
- ◯ Public official
- ◯ Another relationship
- ◯ Refused to answer

A3 Gender of the respondent
- ◯ Male
- ◯ Female

A4 Did you/the respondent live with the deceased in the period leading to his/her death?
- ◯ Yes
- ◯ No

A5 Were you present with the deceased at the time of death
- ◯ Yes
- ◯ No

section_2b_characteristics_of_deceased_child_aged

B0 Give his/her initials ______________________

B1 When was (NAME) born? ______________________
(Enter 01-01-1900 if Unknown)

B2 When did he/she die? ______________________
(Enter 01-01-1900 if Unknown)

B3 Where did the deceased die?	◯ Hospital<br>◯ Other health facility<br>◯ Home<br>◯ Enroute health facility<br>◯ Other(specify)<br>◯ Doesn't know<br>◯ Refused to answer
B3a Other place of death	__________________
B3b Did the family obtain a death certificate for the deceased?	◯ Yes<br>◯ No<br>◯ Don't know<br>◯ Refuse to answer
B4 How many days old was the baby? (enter neonate's age)	__________________<br>(Indicate 99 if respondent does not know)
B5 What was her/his race	◯ Black African<br>◯ White<br>◯ Coloured<br>◯ Indian<br>◯ Asian<br>◯ Other (Specify)
B5a Race if other was indicated	__________________

section_2c_verification_of_possible_stillbirth

C1 Did the baby ever cry?	◯ Yes<br>◯ No<br>◯ Doesn't know<br>◯ Refused to answer
C2 Did the baby cry immediately after birth, even if only a little bit?	◯ Yes<br>◯ No<br>◯ Doesn't know<br>◯ Refused to answer
C3 How many minutes after birth did the baby first cry?	__________________<br>(Indicate 99 if respondent does not know)
C4 Did the baby stop being able to cry?	◯ Yes<br>◯ No<br>◯ Doesn't know<br>◯ Refused to answer
C5 How many hours before death did the baby stop crying?	__________________<br>(Indicate 99 if respondent does not know)

C7 Did the baby ever move?	◯ Yes<br>◯ No<br>◯ Doesn't know<br>◯ Refused to answer
C8 Did the baby ever breathe?	◯ Yes<br>◯ No<br>◯ Doesn't know<br>◯ Refused to answer
C9 Did the baby breathe immediately after birth, even a little?	◯ Yes<br>◯ No<br>◯ Doesn't know<br>◯ Refused to answer
C10 Did the baby have a breathing problem?	◯ Yes<br>◯ No<br>◯ Doesn't know<br>◯ Refused to answer
C11 Was the baby given assistance to breathe at birth?	◯ Yes<br>◯ No<br>◯ Doesn't know<br>◯ Refused to answer
C12 If the baby didn't show any sign of life, was it born dead?	◯ Yes<br>◯ No<br>◯ Doesn't know<br>◯ Refused to answer
C13 Were there any bruises or signs of injury on baby's body after the birth?	◯ Yes<br>◯ No<br>◯ Doesn't know<br>◯ Refused to answer
C14 Was the baby's body soft, pulpy and discoloured and the skin peeling away	◯ Yes<br>◯ No<br>◯ Doesn't know<br>◯ Refused to answer

section_2d_health_history

E1 How many days old was the baby when the fatal illness started?	________________________
E2 Before the illness that led to death, was the baby/child growing normally?	◯ Yes<br>◯ No<br>◯ Doesn't know<br>◯ Refused to answer
E3 For how many days was he/she ill before death?	________________________<br>(Indicate 99 if respondent does not know)
E4 Did he/she die suddenly?	◯ Yes<br>◯ No<br>◯ Doesn't know<br>◯ Refused to answer

E5 Did he/she have a fever?	◯ Yes<br>◯ No<br>◯ Doesn't know<br>◯ Refused to answer
E6 How many days did the fever last?	______________________________<br>(Indicate 99 if respondent does not know)
E7 Did the fever continue until death?	◯ Yes<br>◯ No<br>◯ Doesn't know<br>◯ Refused to answer
E8 Did he/she have a cough?	◯ Yes<br>◯ No<br>◯ Doesn't know<br>◯ Refused to answer
E9 Did he/she make a whooping sound when coughing?	◯ Yes<br>◯ No<br>◯ Doesn't know<br>◯ Refused to answer
E10 Did he/she have any difficulty breathing?	◯ Yes<br>◯ No<br>◯ Doesn't know<br>◯ Refused to answer
E12 How many days did the fast breathing last?	______________________________
E15 Did you see the lower chest wall/ribs being pulled in as the child breathed in?	◯ Yes<br>◯ No<br>◯ Doesn't know<br>◯ Refused to answer
E17 Did he/she have more frequent loose or liquid stools than usual?	◯ Yes<br>◯ No<br>◯ Doesn't know<br>◯ Refused to answer
E18 How many stools did the baby or child have on the day that loose liquid stools were most frequent?	______________________________<br>(Indicate 99 if respondent does not know)
E19 How many days before death did the frequent loose or liquid stool start?	______________________________<br>(Indicate 99 if respondent does not know)
E20 At any time during the final illness was there blood in the stools?	◯ Yes<br>◯ No<br>◯ Doesn't know<br>◯ Refused to answer

E21 Did he/she vomit in the week preceding the death?	◯ Yes<br>◯ No<br>◯ Doesn't know<br>◯ Refused to answer
E22 Was he/she unconscious during the illness that led to death?	◯ Yes<br>◯ No<br>◯ Doesn't know<br>◯ Refused to answer
E23 Was he/she unconscious for more than 24 hours before death?	◯ Yes<br>◯ No<br>◯ Doesn't know<br>◯ Refused to answer
E24 Did he/she have convulsions?	◯ Yes<br>◯ No<br>◯ Doesn't know<br>◯ Refused to answer
E25 During the illness that led to death, did he/she have any skin rash?	◯ Yes<br>◯ No<br>◯ Doesn't know<br>◯ Refused to answer
E26 During the illness that led to death, did he/she have areas of the skin that turned black?	◯ Yes<br>◯ No<br>◯ Doesn't know<br>◯ Refused to answer
E27 During the illness that led to death, did he/she have areas of the skin with redness and swelling?	◯ Yes<br>◯ No<br>◯ Doesn't know<br>◯ Refused to answer
E28 During the illness that led to death, did he/she bleed from anywhere?	◯ Yes<br>◯ No<br>◯ Doesn't know<br>◯ Refused to answer
E29 Did he/she have yellow discolouration of the eyes or skin, palms (hand) or soles (foot) ?	◯ Yes<br>◯ No<br>◯ Doesn't know<br>◯ Refused to answer
E30 Was the baby able to suckle or bottle-feed within the 24 hours after birth?	◯ Yes<br>◯ No<br>◯ Doesn't know<br>◯ Refused to answer
E31 Did the baby stop suckling?	◯ Yes<br>◯ No<br>◯ Doesn't know<br>◯ Refused to answer
E32 How many days after birth did the baby stop suckling?	____________________<br>(Indicate 99 if respondent does not know)

E33 Did the baby have convulsions starting within 24 hours of life?	◯ Yes<br>◯ No<br>◯ Doesn't know<br>◯ Refused to answer
E34 Did the baby's body become stiff, when the back arched backwards?	◯ Yes<br>◯ No<br>◯ Doesn't know<br>◯ Refused to answer
E35 During the illness that led to death, did the baby have a bulging or raised fontanelle?	◯ Yes<br>◯ No<br>◯ Doesn't know<br>◯ Refused to answer<br>(Fontanelle is the soft part of a neonates head usually at the front and back of the head but the one in front is more prominent)
E36 During the illness that lead to death, did the baby have a sunken fontanelle?	◯ Yes<br>◯ No<br>◯ Doesn't know<br>◯ Refused to answer
E37 During the illness that led to death, did the baby become cold to touch?	◯ Yes<br>◯ No<br>◯ Doesn't know<br>◯ Refused to answer
E38 Did the baby have redness or pus drainage from the umbilical cord stump?	◯ Yes<br>◯ No<br>◯ Doesn't know<br>◯ Refused to answer
E40 Did the baby or infant appear to be healthy and then just die suddenly?	◯ Yes<br>◯ No<br>◯ Doesn't know<br>◯ Refused to answer

Neonatal and child history, signs and symptoms

E41 Was the child part of a multiple birth?	◯ Yes<br>◯ No<br>◯ Doesn't know<br>◯ Refused to answer
E42 Is the mother still alive?	◯ Yes<br>◯ No
E43 Did you/the mother receive professional assistance during the delivery?	◯ Yes<br>◯ No<br>◯ Doesn't know<br>◯ Refused to answer
E44 What was the weight (in kilograms) of the deceased at birth?	____________________<br>(To one decimal place. Indicate 9 if respondent does not know)

45 At birth, was the baby smaller than usual (weighing under 2.5kg)?

◯ Yes
◯ No
◯ Doesn't know
◯ Refused to answer

E46 Were there any complications in the late part of pregnancy (defined as the last 3 months, before labour)?

◯ Yes
◯ No
◯ Doesn't know
◯ Refused to answer

E46a Type of complications during late pregnancy

____

E47 Were there any complications during labour or delivery?

◯ Yes
◯ No
◯ Doesn't know
◯ Refused to answer

E47a Type of complication during labour or delivery

____

E48 Was any part of the baby physically abnormal at time of delivery? (e.g. body part too large or too small, additional growth on body)?

◯ Yes
◯ No
◯ Doesn't know
◯ Refused to answer

E48a Which part of the body was physically abnormal?

____

E49 Did the baby have a swelling or defect on the back at the time of birth?

◯ Yes
◯ No
◯ Doesn't know
◯ Refused to answer

E50 How many births, including stillbirths, did the baby's mother have before this baby?

____
(Indicate 99 if respondent does not know, 98 if respondent doesn't want to answer)

E51 Was the baby moving in the last few days before the birth?

◯ Yes
◯ No
◯ Doesn't know
◯ Refused to answer

E52 Did the baby stop moving after labour started?

◯ Yes
◯ No
◯ Doesn't know
◯ Refused to answer

E53 How many hours did labour, and delivery take?

____
(Indicate 99 if respondent does not know, 98 if Refuses to answer)

E54 Was the baby born 24 hours or more after the water broke?	◯ Yes<br>◯ No<br>◯ Doesn't know<br>◯ Refused to answer
E55 Was the delivery normal vaginal, without forceps or vacuum?	◯ Yes<br>◯ No<br>◯ Doesn't know<br>◯ Refused to answer
E56 Did you/the mother receive any vaccinations since reaching adulthood including during this pregnancy?	◯ Yes<br>◯ No<br>◯ Doesn't know<br>◯ Refused to answer
E57 Did you/the mother receive tetanus toxoid vaccine?	◯ Yes<br>◯ No<br>◯ Doesn't know<br>◯ Refused to answer
E58 During labour, did the baby's mother suffer from fever?	◯ Yes<br>◯ No<br>◯ Doesn't know<br>◯ Refused to answer
E59 During the last 3 months of pregnancy, labour or delivery, did you/the baby's mother suffer from high blood pressure?	◯ Yes<br>◯ No<br>◯ Doesn't know<br>◯ Refused to answer
E60 Did you/the baby's mother have diabetes mellitus?	◯ Yes<br>◯ No<br>◯ Doesn't know<br>◯ Refused to answer
E61 During the last 3 months of pregnancy, labour or delivery, did you/the baby's mother suffer from convulsions?	◯ Yes<br>◯ No<br>◯ Doesn't know<br>◯ Refused to answer
E61a How many episodes of convulsions did the mother have?	______________________<br>(Indicate 99 if respondent does not know)
E62 During the last 3 months of pregnancy did you/the baby's mother suffer from blurred vision?	◯ Yes<br>◯ No<br>◯ Doesn't know<br>◯ Refused to answer
E63 Did you/the baby's mother have severe anemia?	◯ Yes<br>◯ No<br>◯ Doesn't know<br>◯ Refused to answer

Health service utilisation

E64 Did the baby receive any treatment for the illness that led to death?	◯ Yes<br>◯ No<br>◯ Doesn't know<br>◯ Refused to answer
E65 Was the treatment received at a hospital or clinic?	◯ Yes<br>◯ No<br>◯ Doesn't know<br>◯ Refused to answer
E66 Did he/she receive oral rehydration salts (ORS)?	◯ Yes<br>◯ No<br>◯ Doesn't know<br>◯ Refused to answer<br>(This is usually given to children who have diarrhea)
E67 Did he/she receive intravenous fluids (drip) treatment?	◯ Yes<br>◯ No<br>◯ Doesn't know<br>◯ Refused to answer
E68 Did he/she receive (or need) a blood transfusion?	◯ Yes<br>◯ No<br>◯ Doesn't know<br>◯ Refused to answer
E69 Did he/she receive (or need) treatment/food through a tube passed through the nose?	◯ Yes<br>◯ No<br>◯ Doesn't know<br>◯ Refused to answer
E70 Did he/she receive/need antiretroviral therapy (ART)?	◯ Yes<br>◯ No<br>◯ Doesn't know<br>◯ Refused to answer
E71 Did he/she have (or need) an operation for the illness?	◯ Yes<br>◯ No<br>◯ Doesn't know<br>◯ Refused to answer
E71a What type of operation was needed or did s/he have?	______________________________<br>(Indicate 99 if respondent does not know)
E72 Did he/she receive any immunisations?	◯ Yes<br>◯ No<br>◯ Doesn't know<br>◯ Refused to answer
E72a Which immunisations were received?	______________________________<br>(Indicate 99 if respondent does not know)

E74 Did a health care worker tell you the cause of death?	◯ Yes<br>◯ No<br>◯ Doesn't know<br>◯ Refused to answer
E75 What did the health care worker say?	____
E76 Has the deceased's biological mother ever been told she had HIV/AIDS by a health worker?	◯ Yes<br>◯ No<br>◯ Doesn't know<br>◯ Refused to answer
E76a How long ago (in years)?	__________________________________<br>(Indicate 99 if respondent does not know)

Background and context

E77 In the final days before death, was the baby taken to a hospital or health facility?	◯ Yes<br>◯ No<br>◯ Doesn't know<br>◯ Refused to answer
E78 Did he/she use motorised transport to get to the hospital or health facility?	◯ Yes<br>◯ No<br>◯ Doesn't know<br>◯ Refused to answer
E78a What type of motorized transport was used?	◯ Ambulance<br>◯ Personal car<br>◯ Taxi<br>◯ Cab<br>◯ Other (specify)
E78b Name of transport if other was indicated	__________________________________
E79 Were there any problems during admission to the hospital or health facility?	◯ Yes<br>◯ No<br>◯ Doesn't know<br>◯ Refused to answer
E79a Explain/name of problems encountered during admission	____
E80 Were there any problems with the way he/she was treated in the hospital or health facility?	◯ Yes<br>◯ No<br>◯ Doesn't know<br>◯ Refused to answer
E80a Describe how the baby was treated at the health facility	____

E81 Were there any problems getting medications or diagnostic tests in the hospital or health facility?

- ◯ Yes
- ◯ No
- ◯ Doesn't know
- ◯ Refused to answer

E82 Does it take more than 2 hours to get to the nearest hospital or health facility from the deceased's household?

- ◯ Yes
- ◯ No
- ◯ Don't know
- ◯ Refuse to answer

E83 In the final days before death, were there any doubts about whether medical care was needed?

- ◯ Yes
- ◯ No
- ◯ Doesn't know
- ◯ Refused to answer

E84 In the final days before death, was traditional medicine used?

- ◯ Yes
- ◯ No
- ◯ Doesn't know
- ◯ Refused to answer

section_2e_contributing_factors_and_strategies

F1 Please tell me the factors that you think contributed to (NAME's) death

____
(PROMPTS: Factors which contributed to his/her poor health, individual factors, family factors, community factors, health facility factors)

F2 Please tell me how you think children could be prevented from dying in future

____
(PROMPTS: Strategies for babies, families, communities, health facilities)

F3 Is there anything else you would like to add?

____
