# Supplementary material for: Engaging community health workers in maternal and infant death identification in Khayelitsha, South Africa: a pilot study
Source: BMC Pregnancy Childbirth. 2020 Nov 26;20:736. doi: 10.1186/s12884-020-03419-4 (PMC7690162; doi:10.1186/s12884-020-03419-4)
Supplement: Supplementary file 3 — Additional file 3. Summary of community-linked death review components. [file 12884_2020_3419_MOESM3_ESM.docx]

***Appendix 1: Summary of community-linked death review components***

| ***Key components*** | ***Approaches*** | ***Activities*** |
| --- | --- | --- |
| 1. Establishing an enabling environment | A review of community, local and national maternal and neonatal death reporting systems.  A review of the community health system linkages to death reporting. | Facilitate sessions with community-based organisations to analyse and document relevant issues and plan and implement involvement in policy activities at appropriate levels. |
|  | Advocacy, communication and social mobilisation. | Conduct a community stakeholder and health system mapping exercise to identify strengths and weaknesses in the reporting systems.  Advocacy plan addressing implementation and improvement of local and national death reporting systems. |
| 2. Ensuring intersectoral collaboration and coordination | Building community linkages, collaboration and coordination. | Identify functional networks, linkages and partnerships between community actors and local and national health departments.  Plan for collaborating for effective coordination and decision making of all actors. |
| 3. Resources and capacity building | Human resources: skills building for CHWs, advocacy actors and local leadership. | Develop capacity building programme.  Continuous training for CHWs conducting the death review.  Community actors should have good knowledge of rights, community health, social environments and barriers to access and develop and deliver an effective death review system. |
|  | Financial resources. | Secure core funding and manage financial resources sustainably.  Financial reporting system that is transparent and timely should be implemented. |
|  | Material resources – infrastructure, information and essential commodities (including medical and other products and technologies). | Functional systems should be in place to forecast, quantify, source, manage and use infrastructure and essential commodities in appropriate and efficient ways. |
| 4. Community health system and service delivery | CHWs activities and health services – delivery, use, quality. | Effective, no blame, high-quality reporting is enabled; and responding interventions are equitably delivered to those women and neonates in order to prevent mortalities. |
| 5. Organizational and leadership strengthening | Management, accountability and leadership. | While ensuring accountability to all stakeholders CHWs must provide leadership in the development, operation and management of the death reporting. |
| 6. Monitoring & evaluation and planning | Monitoring & evaluation, evidence building. | Relevant programmatic qualitative and quantitative data is collected, analyzed, used and shared. Appropriate mechanisms for data quality, feedback and supervision should be in place. |
|  | Strategic and operational planning. | Strategic information generated by the M&E system is used for evidence‐based planning, management, advocacy and policy formulation. |
